# Study of the Effect of Intestinal Microbes on Obesity: A Bibliometric Analysis

**DOI:** 10.3390/nu15143255

**Published:** 2023-07-23

**Authors:** Zehao Su, Chenyu Tian, Guan Wang, Jingjing Guo, Xiaoyan Yang

**Affiliations:** 1 Department of Endocrinology & Metabolism and West China Biomedical Big Data Center, West China Hospital, Sichuan University/West China School of Nursing, Chengdu 610041, China; suzehao@wchscu.cn (Z.S.); tianchenyu@wchscu.cn (C.T.); 2 Med-X Center for Informatics, Sichuan University, Chengdu 610041, China

**Keywords:** obesity, intestinal microbiota, bibliometric analysis, visualization, co-occurrence analysis

## Abstract

Obesity is a serious public health problem. According to statistics, there are millions of obese people worldwide. Research studies have discovered a complex and intricate relationship between the gut microbiota and obesity. Probing and summarizing the relationship between intestinal microbes and obesity has important guiding significance for the accurate control of the research direction and expanding the choice of obesity treatment methods. We used bibliometric analysis to analyze the published literature with the intention to reveal the research hotspots and development trends on the effects of intestinal microbes on obesity from a visualization perspective, both qualitatively and quantitatively. The results showed that current research is focusing on related mechanisms of the effects of intestinal microbes on obesity and therapeutic methods for obesity. Several noteworthy hotspots within this field have garnered considerable attention and are expected to remain the focal points of future research. Of particular interest are the mechanisms by which intestinal microbes potentially regulate obesity through metabolite interactions, as well as the role of microbiomes as metabolic markers of obesity. These findings strongly suggest that gut microbes continue to be a key target in the quest for effective obesity treatments. Co-operation and communication between countries and institutions should be strengthened to promote development in this field to benefit more patients with obesity.

## 1. Introduction

Obesity is a serious public health problem. According to statistics, there are approximately 600 million obese people and 1.9 billion overweight individuals worldwide [1]. It is predicted that the number of obese people in the world will reach 1.12 billion by 2030 [2]. As far as we know, obesity is not only a change in appearance, but it is also associated with disorders of lipid and glucose metabolism, chronic inflammation, oxidative stress, and increased risk of a variety of diseases. According to research, obesity is one of the non-negligible reasons for cardiovascular disease, diabetes, and malignant tumors [3,4], while it is estimated that more than 80% of the global burden of diabetes and cardiovascular disease will occur in developing countries such as China and India in 2025 [5]. However, the etiology of obesity is not yet completely clear. The increasing prevalence of obesity cannot be explained by changes in energy balance alone. Therefore, it is vital for the whole world to be involved in determining the formation mechanism of obesity and then to explore effective methods to treat it in order to reduce the incidence and mortality of obesity-related diseases.

The intestinal microbiota is a microbiota that lives in the gastrointestinal tract of the human body and can interact with the external environment through diet [6]. These microbes colonize the intestinal tract through mother-to-child transmission from the prenatal period. The colonization of human intestinal microbes continues after birth and is regulated by factors including gestational age, mode of delivery (natural delivery or cesarean section), diet (breastfeeding or baby food), hygiene, and antibiotics. The environment and diet of the first three years of life are essential for the acquisition of adult-like microbiota and the establishment of bacteria-host symbiosis that affects the immune and nervous system development. Between the ages of two and five, the intestinal microbiota gradually stabilize, and there is no significant difference with the intestinal microbiota in adults [7]. Under normal circumstances, the intestinal microbiota maintains a symbiotic relationship with the human body, and a good symbiotic relationship between them is very important for the maintenance of human health [8]. However, instantaneous changes in the intestinal ecosystem can lead to the destruction of this symbiotic relationship between microorganisms and hosts, such as aging, obesity, sedentary lifestyles, and dietary patterns, and the effects of antibiotics can change the intestinal microbiota [9]. Because of the important role of the intestinal ecosystem in maintaining host physiology, its changes can also cause a variety of physiological disorders in the human body, including low-grade inflammation, metabolic disorders, excessive lipid accumulation, and loss of insulin sensitivity, in turn leading to changes in the composition of the intestinal microbiota and decreasing the diversity of flora and metabolic pathways [10,11], thus increasing the risk of metabolic diseases such as obesity and diabetes [12]. Probing and summarizing the relationship between intestinal microbes and obesity has important guiding significance for the accurate control of the research direction and expanding the choice of obesity treatment methods.

Bibliometrics is a discipline that uses mathematics and statistics to quantitatively analyze literature and information. Through quantitative analysis of the subject literature, we can construct a knowledge structure and explore the development trend. At present, it has been widely used in many research fields [13,14,15]. Overall, this analysis method and the traditional literature review are both based on previous research, summarizing the current situation and shortcomings of the research, and finally guiding further research. A literature review emphasizes the content of an article, that is, summarizing the aspects and shortcomings of existing research, while most studies quote the representative papers in the literature according to a preset research context. On the other hand, bibliometric analysis places more emphasis on the analysis of “quantity”. There is no need to analyze the research content of each document in detail but to analyze the number of published documents, the distribution of authors, and the relationship of citations in the target field. Most of the included documents are highly quoted. Compared with the traditional literature review, bibliometric analysis can make a more intuitive systematic analysis of all the literature in this field in a visual way, which is helpful for researchers entering a new field to grasp the overall trend of the field. There have been many review articles in the field of intestinal microbes and obesity, but the quantitative analysis in the field is insufficient. Therefore, in addition to briefly summarizing the hotpots in review articles, we used bibliometrics to analyze the published literature with the intention to reveal the research hotspots and development trends in this field, both qualitatively and quantitatively.

## 2. Materials and Methods

### 2.1. Data Sources and Search Strategies

Data were obtained from the core collection of Web of Science (WoSCC), a database of Clarivate Analytics. The following search strategies were presented: ((overweight OR obesity OR obes* OR over$weight) AND (gastrointestin* OR gastro-intestin* OR gut OR intestin*) AND (bacteria OR prebiotic OR probiotic OR microbiot* OR microbiome* OR flora OR microflora)). In the end, a total of 8888 original articles in English were retrieved. All the documents were downloaded as “Full Record and Cited References” and saved as “Bibtex” and “plain text file”. The above data were obtained on 2 February 2023.

### 2.2. Data Analysis and Graph Acquisition

The corresponding files retrieved from WoSCC were imported into Microsoft Excel (16.66.1), Biblioshiny (4.1) and CiteSpace (6.1.R6) to perform a bibliometric and visual analysis. Annual publications, country annual publications, and publisher annual publications were analyzed with Microsoft Excel. The Biblioshiny platform provides a web-based graphical interface in the Bibliometrix package, which was used to conduct total citations and average articles cited, as well as showing publications of journals and a country collaboration map. CiteSpace is an interactive visualization analysis software developed by Professor Chen Chaomei to conduct different types of network analyses, such as country collaboration networks, institution collaboration networks, author collaboration networks, co-cited author collaboration networks, co-cited journal collaboration networks, and keyword co-occurrence, which aid in visually analyzing the knowledge domain and emerging trends. A dual-map overlay was constructed for journals. The parameters of CiteSpace were as follows: time slicing (2013 to 2022), years per slice (1), links (strength: cosine, scope: within slices), selection criteria (g-index: k = 5 in country, institution, author collaboration network analysis and keyword co-occurrence), pruning (pathfinder, pruning sliced networks). Cluster labels used keywords by log likelihood ratio (LLR). To identify emerging topics, we detected keywords and references with a strong citation burst.

Ethical approval was waived by the local ethical board because the data came from public databases, and no human or animal subjects were involved

## 3. Results

### 3.1. An Overview of the Annual Growth Trend

**Figure 1:**
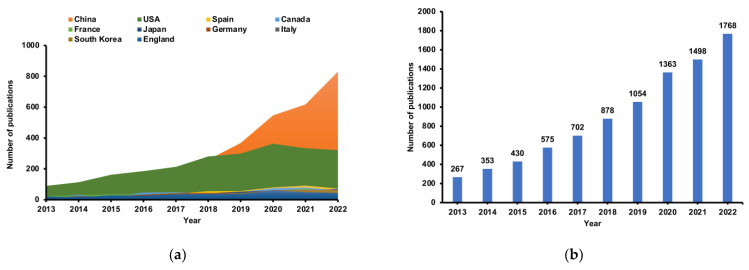

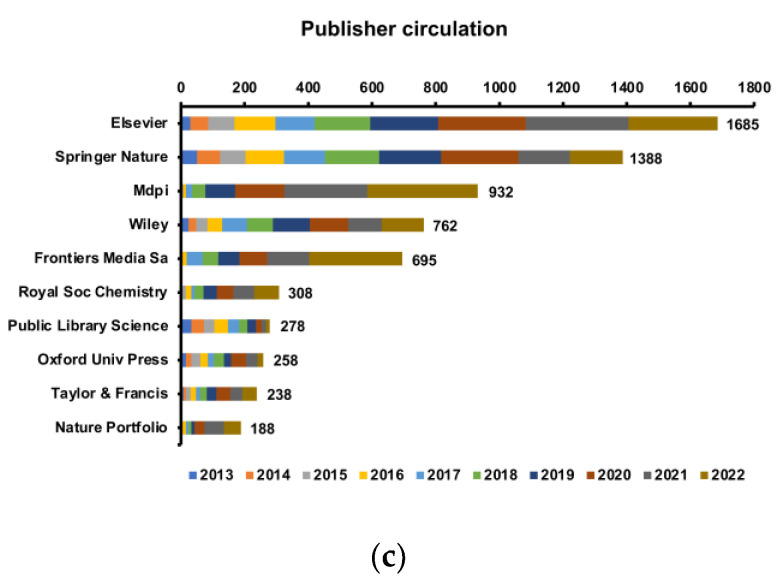
Global publication trend and country or region analysis for the effect of intestinal microbes on obesity research. (**a**) Annual worldwide publication output. (**b**) Growth trends in publication output from the top 10 most productive countries. (**c**) Top 10 publishers according to their contribution to the total number of articles on the effect of intestinal microbes on obesity research.

As shown in Figure 1a, from 2013 to 2022, a total of 8888 English language articles met our inclusion criteria. The number of annual publications began to exceed 500 in 2016, and only after 3 years did the annual publications exceed 1000. The annual number of publications increased from 267 in 2013 to 1768 in 2022, with an amazing increase of over sixfold. We further analyzed the annual national output of the 10 most productive countries and regions (Figure 1b). The different colors represent different countries, and the slope represents the speed of annual publication number increase. China ranked first in the number of annual publications, followed by the United States and Spain. The top 10 publishers according to their contribution to the total number of articles about the effect of intestinal microbes on obesity are shown in Figure 1c. The various colors represent different year publications. Regarding the number of publications, Elsevier (1685) and Springer Nature (1388) far surpassed other publishers, confirming their unsurpassable international position in the publishing industry.

### 3.2. Distribution and Cooperation between Countries/Regions and Institutions 

**Figure 2:**
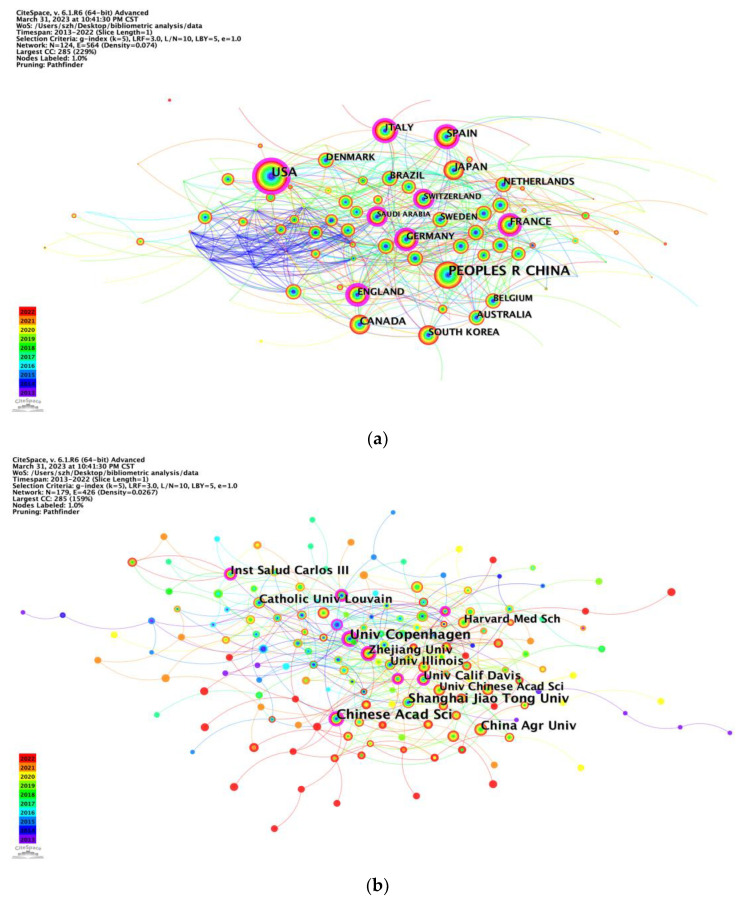
CiteSpace network visualization map of countries/regions and institutions. (**a**) CiteSpace network visualization map of countries/regions involved in the effect of intestinal microbes on obesity research (node label: by citation, label font size: proportional). (**b**) CiteSpace network visualization map of institutions involved in the effect of intestinal microbes on obesity research (node label: by citation, label font size: proportional).

In the ten years from 2013–2022, 124 countries or regions participated in the domain of intestinal microbes and obesity. China ranked first with 3036 publications, accounting for 34.16% of all publications, followed by the United States (2356, 26.51%) and Spain (480, 5.40%) (Table 1, Figure 2a). Nevertheless, the United States tops the ranking of the most cited countries with a frequency (number of documents) of 87,065, followed by China (65,250), France (13,809), and Canada (13,496) (Table S1). The country collaboration map shows the overall perspective of country and region academic cooperation (Figure S1). The United States had the broadest academic associations with other countries or regions, while its collaboration with China was the tightest, followed by Canada. The United States and China both had frequent connection with European countries. Moreover, among European countries, there was also tight collaboration, but communication among the other countries or regions still needs to be strengthened.

There were 179 institutions included in the results of institution analysis. The top 10 institutions mostly came from China (n = 5) and the USA (n = 2), followed by Denmark (n = 1), Belgium (n = 1), and Spain (n = 1). University of Copenhagen, Chinese Academy of Sciences, and Shanghai Jiao Tong University had the highest total link strength, revealing close cooperation with other institutions (Table 2, Figure 2b).

### 3.3. Distribution and Co-Authorship of Authors

**Figure 3:**
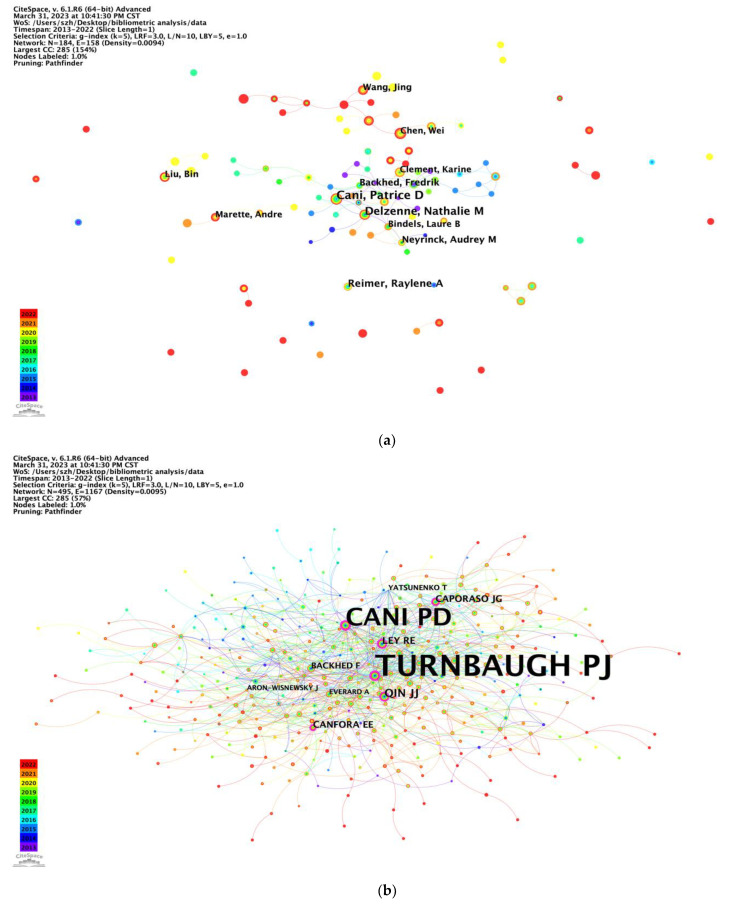
CiteSpace network visualization map of authors and co-cited authors. (**a**) CiteSpace network visualization map of authors involved in the effect of intestinal microbes on obesity research (node label: by citation, label font size: proportional). (**b**) CiteSpace network visualization map of co-cited authors involved in the effect of intestinal microbes on obesity research (node label: by centrality, label font size: proportional).

In this analysis, 16 authors had published at least 21 publications. The 10 most productive authors are listed in Table 3. Most of the authors were from Europe, with Belgium (n = 3), followed by China (n = 3), Canada (n = 2), Sweden (n = 1), and France (n = 1). Patrice D. Cani (97 publications) and Nathalie M. Delzenne (73 publications), both from Université Catholique de Louvain, contributed the top two most publications, followed by Raylene A. Reimer (51 publications) from the University of Calgary. The top two authors maintained the most contact with other research labs, and the cooperation between Patrice D. Cani and Nathalie M. Delzenne was close (Figure 3a). Co-cited authors are shown in Figure 3b and Table 4. Peter J. Turnbaugh and Patrice D. Cani rank as the top two, which manifests their centrality in the research field.

### 3.4. Analysis of Journals and Co-Cited Academic Journals

**Figure 4:**
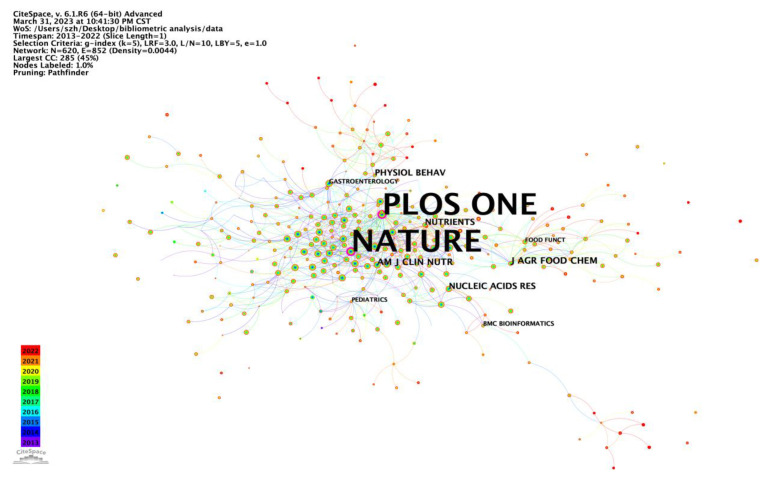
CiteSpace network visualization map of co-cited journals involved in the effect of intestinal microbes on obesity research (node label: by centrality, label font size: proportional).

As shown in Table 5, the top 10 most productive journals published 2155 publications, accounting for 24.25% of all publications. Nutrients (impact factor (IF) 2021, 6.706) published the most articles (399 publications, 4.49%), followed by Scientific Reports (IF 2021, 4.997; 339 publications, 3.81%) and Food & Function (IF 2021, 6.317; 281 publications, 3.16%). The top three highest co-cited journals were Nature (IF 2021, 69.504; 6446 citations), PLOS ONE (IF 2021, 3.752; 6247 citations), and Proceedings of the National Academy of Sciences of the United States of America (PNAS, IF 2021, 12.779; 5329 citations). The co-citation relationship among different journals was visualized in a co-citation network (Table 6, Figure 4). Nature had the highest centrality (0.18), followed by PLOS ONE (0.17), which means that the published articles in the two journals were widely recognized and cited by researchers.

Figure S2 shows the dual-map overlay of journals. The dual-map overlay shows seven main citation paths. The published articles were mostly focused on journals in the fields of medicine, medical, clinical, molecular, biology, and immunology and partly focused on veterinary, animal, and science. Cited articles were mostly published in journals in the fields of molecular biology and genetics, and many articles were published in journals in the fields of health, nursing, medicine, environmental, toxicology, and nutrition.

### 3.5. Analysis of Keywords

#### 3.5.1. Co-Occurrence Analysis

**Figure 5:**
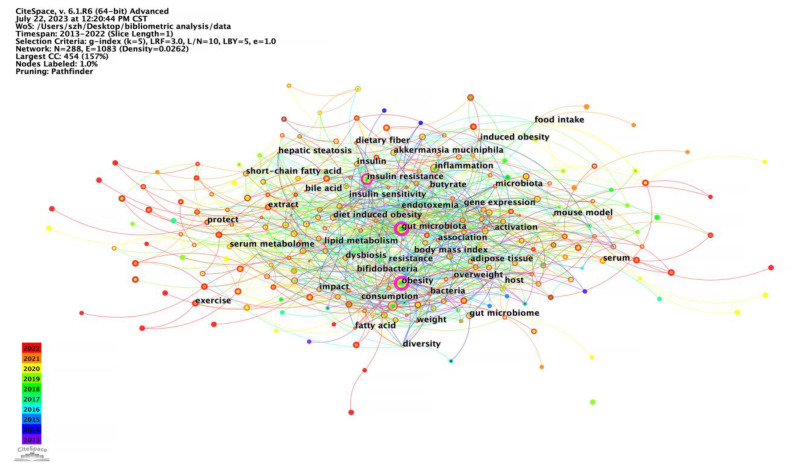
Keywords co-occurrence network for the effect of intestinal microbes on obesity research (node label: by centrality, label font size: uniformed).

Keywords could accurately reflect the main research point of an article, which has high condensation in a research field and can directly point to the center of the text. Therefore, a high frequency of keywords represents hot issues in a research field and research hotspots. The keyword co-occurrence graph for the effects of intestinal microbes on obesity is shown in Figure 5. The density value was 0.0457. The ten most frequent keywords were gut microbiota, obesity, inflammation, insulin resistance, intestinal microbiota, health, metabolism, diet, gut microbiome, and metabolic syndrome (Table 7).

#### 3.5.2. Cluster Analysis

**Figure 6:**
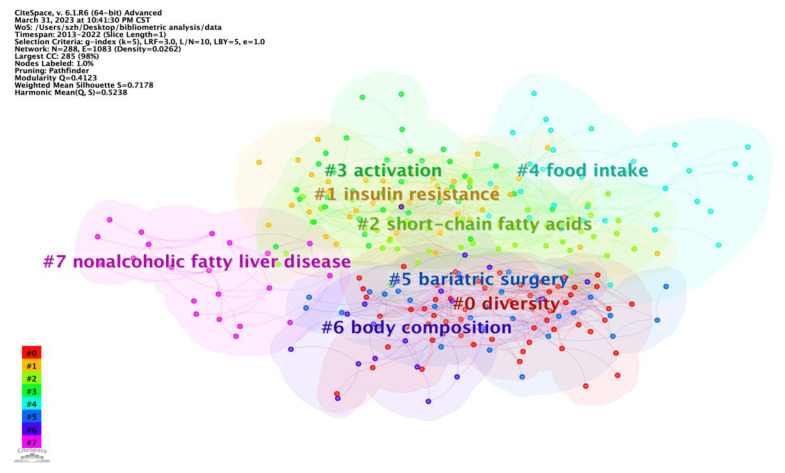
Keywords clusters for the effect of intestinal microbes on obesity research.

The clustering analysis of the keywords yielded 288 nodes and 1083 links, with a Q value of 0.4123 (>0.3) and an S value of 0.7178 (>0.5), which are two important indicators to evaluate the significance of a clustering effect. There were eight clusters, including diversity, insulin resistance, short-chain fatty acids, activation, food intake, bariatric surgery, body composition, and nonalcoholic fatty liver disease (Figure 6). “Diversity” #0 was the largest cluster, followed by “insulin resistance” #1 and “short-chain fatty acids” #2. Timeline View analysis was conducted to further analyze the keywords of the effects of intestinal microbes on obesity (Figure S3).

#### 3.5.3. Burst Detection

CiteSpace was further used to perform burst detection with high frequency and therefore revealed research frontiers and hotspot trends over a period. As shown in Table S2, the first 25 keywords were sorted by strengths of burst to discover the research hotspots about the effects of intestinal microbes on obesity. Keywords such as “ecology” (strength 28.33), “human gut microbiota” (strength 27.34), “microbiota” (strength 23.44), “diet induced obesity” (strength 21.12), and “irritable bowel disease” (strength 16) were the strongest keywords leading the research boom from 2013–2018. Keywords such as “obese” (strength 9.3) and “serum” (9.69) were the most recent trending keywords. Table S3 shows the top 25 references with the strongest citation bursts, which were recognized as a critical milestone and led the development direction of the field for a while.

## 4. Discussion

In this study, we performed a bibliometric analysis of the effects of intestinal microbes on obesity-related studies from 2013 to 2022 using the core collection of WoSCC to comprehensively understand global research trends and hotspots and provide references for researchers in this field or those who want to become involved in the field.

### 4.1. Global Trends in Effects of Intestinal Microbes on Obesity

The amount of annual scientific production is an important indicator of development in an academic field. In our study, a total of 8888 original articles from 1392 journals met the inclusion criteria. The article annual growth rate reached 22.93%, and the annual growth rate from 2019 to 2020 was the highest, shedding light on the increasing attention and expanding research exploration dedicated to this field. 

The closeness of collaboration between countries/regions, institutions, authors, and journals was assessed, which can help to find the laws of scientific research cooperation, guiding more effective scientific research activities, and promoting potential collaborative opportunities for other groups. 

According to Table S1, the total number of publications in the USA (87,065) was greater than that in China (65,250) and far greater than that in France (13,809), Canada (13,496), and the other top 10 most cited countries. The total citations of the United States and China are far more than the other top 10 most cited country citations combined. Without doubt, the United States and China demonstrate their position as leaders in the field. Notably, Israel, with 49 publications, was the most cited country per article (average article citations, 124.18), suggesting that the quality of research on the effects of intestinal microbes on obesity in Israel is very high. A similar story is unfolding in many European countries such as Belgium (average article citations, 107.85) and Sweden (average article citations, 103.95), etc., which sets a model for other countries/regions in this research field (Tables S1 and S4). To improve the quality of articles, the reliability of the data source and the rigor of the experimental design may be the first factors affecting the quality of publications, and more attention should be given to these aspects in future studies.

According to Figure 2b, the bottom right is most dominated by universities from China, while the upper left is most dominated by schools in North America or Europe. Nevertheless, more cooperation between the two clusters could be conducted to catalyze breakthrough progress in research on the effects of intestinal microbes on obesity. Four of the top 10 institutions are based in China, followed by the United States with three, which are thereby maximizing regional advantages and demonstrating the dominance of the United States and China in the field. This may partly explain why China and the United States consistently maintain a high quantity of publications. The University of Copenhagen in Denmark was the most productive institution worldwide, followed by the Chinese Academy of Sciences in China, indicating that these two institutions participated in the most collaborations with other institutions worldwide. Although Spain and Canada ranked third and fourth in terms of total publications, only one of the Spanish research institutions ranked in the top 10, indicating a lack of institutions with professional and research stature in terms of the effects of intestinal microbes on obesity research (Table 1 and Table 2). The most effective organizations and groups are leading the trends on the effects of intestinal microbes on obesity research; thus, further study at these institutions will ensure continuous future development in this field.

Notably, Prof. Patrice D. Cani from Université Catholique de Louvain, Belgium, has published the most articles with the highest centrality, mainly focusing on physiology, molecular metabolism, and nutrition. Researchers have emphasized the significant roles played by the intestinal microbiota in the development of diseases associated with overweight and obesity, including type 2 diabetes, cardiovascular diseases, and certain cancers. Prof. Patrice D. Cani discovered a very particular bacterium called *Akkermansia muciniphila*, which has beneficial effects on health by strengthening the intestinal barrier, decreasing body weight and fat mass gain while decreasing insulin resistance and diabetes [16]. Recently, his team discovered a new bacterium called *Dysosmobacter welbionis*, a completely new genus isolated from the human intestine. Interestingly, they found that this bacterium was present in the intestinal microbiota of the general population but was less abundant in the intestines of individuals with obesity and type 2 diabetes. Experimental studies have demonstrated that the administration of this bacterium improves the health of obese and diabetic mice [17]. The most cited co-author, Peter J. Turnbaugh, is from the University of California San Francisco, United States. He employed interdisciplinary approaches utilizing preclinical models and human cohorts to investigate the mechanisms by which the gut microbiome influences nutrition and pharmacology. His team observed that the consumption of diets exclusively composed of animal or plant products led to rapid changes in the structure of the gut microbial community. These changes were significant enough to override any pre-existing inter-individual differences in microbial community gene expression [18]. This research finding was published in the prestigious journal Nature and has become the most highly cited article in the field.

Conducting an analysis of the characteristics of international peer-reviewed journals enables us to gain insights into current research directions and keep pace with the cutting-edge research in the field. Among the top 10 influential journals in the field of intestinal microbe effects on obesity research, three of them (Nutrients, Scientific reports, PLOS ONE) were also among the top 10 co-cited journals. Undoubtedly, Nutrients, Scientific Reports, Food & Function, and PLOS ONE have emerged as the leading publications, publishing the most relevant articles and maintaining their popularity among researchers who wish to stay updated on the latest research trends. Notably, three of the top 10 co-cited references were published in Nature, one in PNAS, and one in Gut (Table 8). Remarkably, Nature had an impact factor (IF) of 69.504 (2021), followed by Gut (IF, 31.795) and PNAS (IF, 12.779), underscoring their significant contributions in the field of intestinal microbe effects on obesity research.

### 4.2. Basic Knowledge of the Effects of Intestinal Microbes on Obesity Research

The most frequently cited references possess a pivotal academic position in the field. Either positive or negative conclusions from the documents influence the research direction of others. In our study, the top co-cited references were used to investigate the knowledge base for the effects of intestinal microbes on obesity research. The top 10 co-cited references consist of six articles, two letters, one correspondence, and one brief communication [16,18,19,20,21,22,23,24,25,26]. Among them, two articles and one letter experimentally demonstrate that *A. muciniphila* could decrease body weight and fat mass gain while decreasing insulin resistance and diabetes, three articles and one letter focused on omics studies to uncover the mechanisms of the interactions between microorganisms and their hosts, one correspondence and one brief communication developed software and data analysis platforms to make more accurate and full use of sequencing data, and one article experimentally demonstrated the transmissibility of diet-by-microbiota interactions. Therefore, combining microbial sequencing with metabolomics analysis to examine changes in microbial metabolites is a widely employed research approach in this field. *A. muciniphila* has attracted significant attention for its impact on human obesity and its potential for obesity treatment. The impact of diet on gut microbiota remains a focal point of research (Table 8).

### 4.3. Current Hotspots and Field Development Predictions of the Effects of Intestinal Microbes on Obesity Research

Keywords with high frequency are usually used to accurately reveal the main research interests and hotspots in the field within a certain period. Combined with burst detection, which helps researchers to precisely catch up to the research trends from numerous studies, could effectively capture the dramatic increases in references or keywords in one research field within a specified period. Therefore, it served as an important indicator of research hotspots or research frontiers over time (Table S2). The “obese patient” and “serum” burst separately in 2019 and 2020 and continue to burst until now, which indicated that the two topics have received continuous attention in recent years and might be the main trends of research on the effect of intestinal microbes on obesity. During the co-citation reference burst value analysis, five references stood out prominently, focusing primarily on key bacterial metabolites such as short-chain fatty acids, succinate, and secondary bile acids. These references also discuss the benefits of *A. muciniphila* supplementation in improving metabolic parameters in obese patients. Additionally, these references present the latest tools for microbiome data analysis, which warrants thorough exploration and investigation (Table S3). In conjunction with the keywords that continue to exhibit high burst values, we conducted an analysis of the current research hotspots and identified future research trends.

#### 4.3.1. Intestinal Microbes May Regulate Obesity through Bacterial Metabolites

There are complex interactions between the host and intestinal microbes in carbohydrate, amino acid, lipid, and nucleic acid metabolism. Intestinal microbes can use their respective metabolites to maintain intestinal viability while affecting the development, homeostasis and function of the host immune system through nutrition- and metabolite-dependent mechanisms [27]. Small molecules, such as vitamins, fatty acids, amino acids, and bile acids, regulate host-intestinal metabolic homeostasis by binding to specific host membranes or nuclear receptors [28,29]. Short-chain fatty acids (SCFAs), as one of the major products from microbial fermentative activity in the gut [30], can directly activate G protein-coupled receptors, inhibit histone deacetylases, and serve as energy substrates [31,32]. G protein-coupled receptors are also called free fatty acid receptors (FFARs) since they sense free fatty acids. SCFAs facilitate gut–brain axis signaling by activating FFAR2 and FFAR3. This leads to increased satiety and reduced food intake, ultimately impacting the host’s body weight [33,34] and other physiological responses, such as immunity, intestinal transit time, and inflammation [35,36,37,38].

Bile acids, such as cholic acid and chenodeoxycholic acid, can facilitate dietary fat and fat-soluble vitamin absorption. Modified bile acids are referred to as secondary bile acids and include deoxycholic acid, lithocholic acid, and ursodeoxycholic acid [39]. Bile acids can directly and rapidly affect the metabolism of bacteria, including membrane damage and disruption of amino acid, nucleotide and carbohydrate metabolism, while short-term exposure to bile acids significantly affects host metabolism by altering the bacterial community structure [40]. Research has found that supplementation with *Parabacteroides distasonis* in mice can modulate the composition of bile acids in the gut, resulting in decreased weight gain, reduced hyperglycemia, and alleviated hepatic steatosis in ob/ob and high-fat diet (HFD)-fed mice [41].

According to the above analysis, although notable advances in the effects of intestinal microbes on obesity have been made, our understanding of the interrelationships between them remains descriptive, and we still have numerous gaps to fill.

#### 4.3.2. Microbiomes May Act as Metabolic Markers of Obesity 

Human obesity is a heterogeneous condition in the context of pathogenesis, pathophysiology, and therapeutic responsiveness. Studies of alterations in the genome—the microbial gut metagenome—may define subsets of adult individuals with different metabolic risk profiles, which could contribute to resolving some of the heterogeneity associated with adiposity-related phenotypes [21]. With the help of sequencing, recent research found that the richness of the human gut microbiome correlated with human metabolic markers. Individuals with a low bacterial richness were characterized by more marked overall adiposity, insulin resistance, and dyslipidemia as well as a more pronounced inflammatory phenotype when compared with high bacterial richness individuals [21]. Correspondingly, higher gut microbiome gene richness and *A. muciniphila* abundance could exhibit the healthiest metabolic status, particularly in fasting plasma glucose, plasma triglycerides, and body fat distribution [19]. Research has also proven that transmissible and modifiable interactions between diet and microbiota influence host biology. The transformation correlated with invasion of members of *Bacteroidales* from Ln into Ob microbiota that prevented development of increased adiposity and body mass in Ob cage mates and transformed their microbiota metabolic profile to a lean-like state. Therefore, it may be possible to intervene in obesity by targeting the gut microbiota [22]. The gut microbiome can also rapidly respond to an altered diet, and a dynamic balance can be achieved between intestinal microbes and diet immediately, indicating that not only a long-term diet but also a short-term diet can influence the gut microbiome [18]. 

All these findings suggest that gut microbiome richness is a key factor in maintaining the homeostasis of human health and may act as a metabolic marker of obesity

#### 4.3.3. Gut Microbes Could Be a Target for Obesity Treatment

Bariatric surgery is considered the only effective and sustainable weight loss method for obese patients. There is a 50–70% decrease in body weight and fat mass after surgical procedures such as Roux-Y gastric bypass (RYGB) and sleeve gastrectomy (SG) [42]. Nevertheless, obese patients undergoing bariatric surgery may experience overgrowth of small intestinal bacteria [43], a condition that snags with weight loss and increases the risk of micronutrient deficiency that appears to be harmful for the configuration and composition of intestinal microbiota [44,45]. To maintain the effect of surgery and avoid weight rebound, it is important for obese patients to correct the microbial balance and improve microbiota-host interactions with specific interventions [46]. Research has also shown that the elevated pH resulting from RYGB could ensure the survival of probiotic bacteria, making it possible for surgical patients to receive probiotic therapy (80). Probiotics are a kind of active microorganism beneficial to the host that colonizes the human intestinal tract and reproductive system, and they can improve the host microecological balance and play a beneficial role. Bariatric surgery is often followed by an increase in *Streptococcaceae* and a decline in *Bifidobacteriaceae* [42]. Proper supplementation with probiotics can compensate for the intestinal microbial imbalance caused by surgery. Moreover, recent research showed that probiotic intervention could increase the levels of peptide YY and GLP-1 in mice [47], reduce the level of intestinal inflammation [48], and induce the production of anti-inflammatory cytokines [49]. Meanwhile, intestinal peptide signals activate the gut–brain axis, which, in turn, exerts endocrine effects on other organ systems, especially the brain, regulating appetite, metabolism, and other dietary behaviors [33,34,50,51]. Consequently, weight loss and reduced insulin resistance occur. 

Weight-loss intervention by bariatric surgery partially reversed obesity-associated microbial and metabolic alterations in obese individuals [26]. This means that external interventions can affect intestinal microbes. It may be possible to intervene in obesity by targeting the gut microbiota [22], and there is already evidence proving this speculation. *A. muciniphila* is a mucin-degrading bacterium that resides in the mucus layer. A research study found that *A. muciniphila* decreased in obese and type 2 diabetic mice, and *A. muciniphila* treatment reversed high-fat diet-induced metabolic disorders, including fat-mass gain, metabolic endotoxemia, adipose tissue inflammation, and insulin resistance [16]. Pasteurized *A. muciniphila* could enhance its capacity to reduce fat mass development, insulin resistance, and dyslipidemia. Moreover, Amuc_1100, a purified membrane protein from *A. muciniphila*, could improve the gut barrier and partly recapitulate the beneficial effects of the bacterium [23]. All these findings are of great driving significance, indicating that it is feasible to use microbes to treat obesity, but more research is needed to support this hypothesis. However, due to the heterogeneity of experimental techniques, methods, and objects, the effectiveness and safety of microbial product intervention in improving obesity against intestinal microbiota need to be further verified, and the mechanism remains unclear. Few studies have focused on the development of new functional microbiological products, and most of them have been carried out in mice. Before microbial products can be reasonably and effectively used as treatment for obesity, a large number of studies are needed, especially randomized controlled clinical trials.

#### 4.3.4. New Technology Promotes In-Depth Research on the Role of Intestinal Microbes in Obesity

Among the top 10 co-cited references, we found that second-generation sequencing was a vital technology that relies on its high-throughput characteristics, making it easy to sequence the transcriptome or genome of a species [52]. With the development of sequencing technology, a large amount of data is generated. To make more accurate and full use of sequencing data, software and data analysis platforms have also been developed. An open-source software package named DADA2 was used for modeling and correcting Ill umina-sequenced amplicon errors. With the help of this software, researchers could accurately reconstruct amplicon-sequenced communities at the highest resolution, which ensured the accuracy of the research to the greatest extent [24]. Another utility tool named QIIME 2 could serve not only as a marker-gene analysis tool but also as a multidimensional and powerful data science platform that can be rapidly adapted to analyze diverse microbiome features [25]. These tools help to drive rapid development in microbiome research (Table 8). The serum metabolome has emerged as a technique that focuses on defining the functional status of host–microbial relationships in biological specimens, which can reflect the dynamic changes in metabolites and explore disease-related metabolites or dysregulated metabolic pathways (metabolome analysis for investigating host–gut microbiota interactions). A top 10 co-cited reference showed the gut microbiome and serum metabolome alterations in obesity that patients after bariatric surgery not only appeared partially to reverse obesity-associated microbial alterations but also were accompanied by metabolic alterations, including the decreased abundance of *Bacteroides thetaiotaomicron* and the elevated serum glutamate concentration, which also suggests that it may be possible to intervene in obesity by targeting the gut microbiota [26]. 

### 4.4. Strengths and Limitations

This bibliometric study conducted a systematic analysis of the basic situation, research hotspots, and trends in effects of intestinal microbes on obesity from a visualization perspective. The results of the bibliometric study were objective and accurate, which could provide a comprehensive guide for academics who are already working or wish to work in this field. Nevertheless, there are still some limitations in our study. First, owing to the nature of the CiteSpace software, our data are filtered only from the WoSCC database, which is not sufficiently comprehensive and may lead to data omission. Second, our results were processed by CiteSpace software with certain algorithms, which could lead to bias in some of the results. Finally, only English articles were included from the database and analysis, potentially leading to a source bias.

## 5. Conclusions

The effect of intestinal microbes on obesity was investigated by bibliometric analysis in the current study over the period of 2013 to 2022. Compared with other review articles, the contribution of the study is evident in its visualized ways of revealing the countries/regions, institutions, co-authorship of authors, journals and co-cited journals, and popular keywords and references that exert great influence in research on the effect of intestinal microbes on obesity. Our study highlights the potential role of intestinal microbes in regulating obesity through metabolite interactions and identifies microbiomes as potential metabolic markers for obesity. While bariatric surgery is widely recognized as the most effective and sustainable weight loss method for obese patients, our findings suggest that targeting gut microbes could also hold promise as a future approach to obesity treatment. Nevertheless, cooperation and communication between countries and institutions should be strengthened to promote development in this field and to benefit more patients with obesity.

## Figures and Tables

**Table 1:** Top 10 productive countries/regions related to the effect of intestinal microbes on obesity.

Rank	Countries	Articles	Centrality	Percentage (n/8888)
1	China	3036	0.02	34.16%
2	the United States	2356	0.25	26.51%
3	Spain	480	0.14	5.40%
4	Canada	466	0.07	5.24%
5	France	432	0.11	4.86%
6	Japan	406	0.02	4.57%
7	Germany	382	0.12	4.30%
8	Italy	372	0.12	4.19%
9	South Korea	364	0.05	4.10%
10	England	355	0.23	3.99%

**Table 2:** Top 10 productive institutions related to the effect of intestinal microbes on obesity.

Rank	Institutions	Articles	Centrality	Country
1	University of Copenhagen	209	0.28	Denmark
2	Chinese Academy of Sciences	202	0.18	China
3	Shanghai Jiao Tong university	174	0.09	China
4	China Agricultural University	129	0.04	China
5	Catholic University of Louvain	114	0.08	Belgium
6	Zhejiang University	107	0.14	China
7	Inst Salud Carlos III	94	0.10	Spain
8	University of California, Davis	93	0.11	the United States
9	University of Illinois	91	0.09	the United States
10	University of Chinese Academy of Sciences	90	0.02	China

**Table 3:** Top 10 productive authors related to the effect of intestinal microbes on obesity.

Rank	Name	Articles	Centrality	Country
1	Patrice D. Cani	97	0.03	Belgium
2	Nathalie M. Delzenne	73	0.03	Belgium
3	Raylene A. Reimer	51	0.00	Canada
4	Fredrik Backhed	35	0.06	Sweden
5	Audrey M. Neyrinck	34	0.01	Belgium
6	Jing Wang	33	0.00	China
7	Bin Liu	32	0.00	China
8	Andre Marette	28	0.01	Canada
9	Wei Chen	28	0.01	China
10	Karine Clement	27	0.01	France

**Table 4:** Top 10 co-cited authors related to the effect of intestinal microbes on obesity.

Rank	Cited Authors	Articles	Centrality	Country
1	Peter J. Turnbaugh	3307	0.33	United States
2	Patrice D. Cani	2879	0.27	Belgium
3	Ruth E. Ley	2508	0.11	United States
4	Fredrik Backhed	1660	0.09	Sweden
5	J. Gregory Caporaso	1448	0.1	United States
6	Amandine Everard	1370	0.08	Belgium
7	Junjie Qin	1250	0.12	China
8	Robert C. Edgar	935	0.01	United States
9	Nicola Segata	806	0.01	Italy
10	Emmanuelle Le Chatelier	751	0.01	France

**Table 5:** Top 10 productive journals related to the effect of intestinal microbes on obesity.

Sources	Articles	Percentage (n/8888)	IF
Nutrients	399	4.49%	6.706
Scientific reports	339	3.81%	4.997
Food & Function	281	3.16%	6.317
PLOS ONE	265	2.98%	3.752
Frontiers in microbiology	226	2.54%	6.064
Journal of functional foods	171	1.92%	5.223
Molecular nutrition & food research	136	1.53%	6.575
Frontiers in nutrition	116	1.31%	6.590
Journal of agricultural and food chemistry	114	1.28%	5.895
International journal of molecular sciences	108	1.22%	6.208

**Table 6:** Top 10 co-cited journals related to the effect of intestinal microbes on obesity.

Rank	Co-Cited Journals	Articles	Centrality	IF
1	Nature	6446	0.18	69.504
2	PLOS ONE	6247	0.17	3.752
3	Proceedings of the national academy of sciences (PNAS)	5329	0.02	12.779
4	Gut	4389	0.01	31.795
5	Science	4180	0.01	63.832
6	Diabetes	4032	0.02	9.305
7	Scientific reports	3979	0.01	4.997
8	Cell	3308	0.01	66.850
9	Nutrients	3250	0.05	6.706
10	Gastroenterology	3114	0.04	33.883

**Table 7:** Top 10 keywords related to the effect of intestinal microbes on obesity.

Rank	Keywords	Articles	Centrality
1	gut microbiota	4673	0.39
2	obesity	3587	0.23
3	inflammation	1578	0.04
4	insulin resistance	1368	0.12
5	intestinal microbiota	1038	0.01
6	health	869	0.02
7	metabolism	862	0.02
8	diet	848	0.01
9	gut microbiome	825	0.01
10	metabolic syndrome	714	0.01

**Table 8:** The top 10 co-cited references related to the effect of intestinal microbes on obesity.

Rank	Title	Author	Article Type	Journal	DOI	Articles
1	Diet rapidly and reproducibly alters the human gut microbiome	Lawrence et al. (2013) [18]	Letter	Nature	10.1038/nature12820	383
2	Cross-talk between *Akkermansia muciniphila* and intestinal epithelium controls diet-induced obesity	Amandine et al. (2013) [16]	Article	PNAS	10.1073/pnas.1219451110	367
3	*Akkermansia muciniphila* and improved metabolic health during a dietary intervention in obesity-relationship with gut microbiome richness and ecology	Maria et al. (2016) [19]	Article	Gut	10.1136/gutjnl-2014-308778	303
4	A metagenome-wide association study of gut microbiota in type 2 diabetes	Junjie et al. (2012) [20]	Article	Nature	10.1038/nature11450	291
5	Richness of human gut microbiome correlates with metabolic markers	Emmanuelle et al. (2013) [21]	Article	Nature	10.1038/nature12506	285
6	Gut microbiota from twins discordant for obesity modulate metabolism in mice	Vanessa et al. (2013) [22]	Article	Science	10.1126/science.1241214	276
7	A purified membrane protein from *Akkermansia muciniphila* or the pasteurized bacterium improves metabolism in obese and diabetic mice	Hubert et al. (2017) [23]	Letters	Nature Medicine	10.1038/nm.4236	275
8	DADA2: High-resolution sample inference from Illumina amplicon data	Benjamin et al. (2016) [24]	Brief communications	Nature methods	10.1038/nmeth.3869	273
9	Reproducible, interactive, scalable and extensible microbiome data science using QIIME 2	Evan et al. (2019) [25]	Correspondence	Nature biotechnology	10.1038/s41587-019-0209-9	265
10	Gut microbiome and serum metabolome alterations in obesity and after weight-loss intervention	Ruixin et al. (2017) [26]	Article	Nature medicine	10.1038/nm.4358	264

## Data Availability

The data used to support the findings are available from the corresponding author upon request.

## Supplementary Materials

The following supporting information can be downloaded at: https://www.mdpi.com/article/10.3390/nu15143255/s1, Table S1: Top 10 most local cited countries/regions related to the effect of intestinal microbes on obesity, Table S2: Top 25 burst keywords in articles related to the effect of intestinal microbes on obesity, Table S3: Top 25 burst references in articles related to the effect of intestinal microbes on obesity, Table S4: Top 10 most cited countries/regions related to the effect of intestinal microbes on obesity, Figure S1: Country collaboration map. The red line indicates that there was collaboration between two countries, Figure S2: Dual-map overlay related to the effect of intestinal microbes on obesity, Figure S3: Cluster timeline view map of keyword analysis for the effect of intestinal microbes on obesity research.

## References

[1] El-Sayed Moustafa J.S., Froguel P. (2013). From obesity genetics to the future of personalized obesity therapy. Nat. Rev. Endocrinol..

[2] Kelly T., Yang W., Chen C.-S., Reynolds K., He J. (2008). Global burden of obesity in 2005 and projections to 2030. Int. J. Obes..

[3] Boccellino M., Di Domenico M., Donniacuo M., Bitti G., Gritti G., Ambrosio P., Quagliuolo L., Rinaldi B. (2018). AT1-receptor blockade: Protective effects of irbesartan in cardiomyocytes under hypoxic stress. PLoS ONE.

[4] Scherer P.E., Hill J.A. (2016). Obesity, Diabetes, and Cardiovascular Diseases: A Compendium. Circ. Res..

[5] Mohan I., Gupta R., Misra A., Sharma K.K., Agrawal A., Vikram N.K., Sharma V., Shrivastava U., Pandey R.M. (2016). Disparities in Prevalence of Cardiometablic Risk Factors in Rural, Urban-Poor, and Urban-Middle Class Women in India. PLoS ONE.

[6] Federico A., Dallio M., Di Sarno R., Giorgio V., Miele L. (2017). Gut microbiota, obesity and metabolic disorders. Minerva Gastroenterol..

[7] Rodríguez J.M., Murphy K., Stanton C., Ross R.P., Kober O.I., Juge N., Avershina E., Rudi K., Narbad A., Jenmalm M.C. (2015). The composition of the gut microbiota throughout life, with an emphasis on early life. Microbes Ecol. Health Dis..

[8] Jandhyala S.M., Talukdar R., Subramanyam C., Vuyyuru H., Sasikala M., Nageshwar Reddy D. (2015). Role of the normal gut microbiota. World J. Gastroenterol..

[9] Nazli A., Yang P.-C., Jury J., Howe K., Watson J.L., Söderholm J.D., Sherman P.M., Perdue M.H., McKay D.M. (2004). Epithelia Under Metabolic Stress Perceive Commensal Bacteria as a Threat. Am. J. Pathol..

[10] Halmos T., Suba I. (2016). Physiological patterns of intestinal microbiota. The role of dysbacteriosis in obesity, insulin resistance, diabetes and metabolic syndrome. Orv. Hetil..

[11] Sidhu M., van der Poorten D. (2017). The gut microbiome. Austral. Fam. Phys..

[12] Tang W.W., Kitai T., Hazen S.L. (2017). Gut Microbiota in Cardiovascular Health and Disease. Circ. Res..

[13] Gao K., Dou Y., Lv M., Zhu Y., Hu S., Ma P. (2021). Research hotspots and trends of microRNA in periodontology and dental implantology: A bibliometric analysis. Ann. Transl. Med..

[14] Liu Z., Gao K., Hai Y., Liu T. (2021). Developments, Focuses, and Trends in Early-Onset Scoliosis From 2005 to 2020: A Systematic Bibliometric Analysis. World Neurosurg..

[15] Wang Z., Ma D., Pang R., Xie F., Zhang J., Sun D. (2020). Research Progress and Development Trend of Social Media Big Data (SMBD): Knowledge Mapping Analysis Based on CiteSpace. ISPRS Int. J. Geo-Inf..

[16] Everard A., Belzer C., Geurts L., Ouwerkerk J.P., Druart C., Bindels L.B., Guiot Y., Derrien M., Muccioli G.G., Delzenne N.M. (2013). Cross-talk between *Akkermansia muciniphila* and intestinal epithelium controls diet-induced obesity. Proc. Natl. Acad. Sci. USA.

[17] Le Roy T., de Hase E.M., Van Hul M., Paquot A., Pelicaen R., Régnier M., Depommier C., Druart C., Everard A., Maiter D. (2021). *Dysosmobacter welbionis* is a newly isolated human commensal bacterium preventing diet-induced obesity and metabolic disorders in mice. Gut.

[18] David L.A., Maurice C.F., Carmody R.N., Gootenberg D.B., Button J.E., Wolfe B.E., Ling A.V., Devlin A.S., Varma Y., Fischbach M.A. (2014). Diet rapidly and reproducibly alters the human gut microbiome. Nature.

[19] Dao M.C., Everard A., Aron-Wisnewsky J., Sokolovska N., Prifti E., Verger E.O., Kayser B.D., Levenez F., Chilloux J., Hoyles L. (2016). Akkermansia muciniphila and improved metabolic health during a dietary intervention in obesity: Relationship with gut microbiome richness and ecology. Gut.

[20] Qin J., Li Y., Cai Z., Li S., Zhu J., Zhang F., Liang S., Zhang W., Guan Y., Shen D. (2012). A metagenome-wide association study of gut microbiota in type 2 diabetes. Nature.

[21] Le Chatelier E., Nielsen T., Qin J., Prifti E., Hildebrand F., Falony G., Almeida M., Arumugam M., Batto J.-M., Kennedy S. (2013). Richness of human gut microbiome correlates with metabolic markers. Nature.

[22] Ridaura V.K., Faith J.J., Rey F.E., Cheng J., Duncan A.E., Kau A.L., Griffin N.W., Lombard V., Henrissat B., Bain J.R. (2013). Gut Microbiota from Twins Discordant for Obesity Modulate Metabolism in Mice. Science.

[23] Plovier H., Everard A., Druart C., Depommier C., Van Hul M., Geurts L., Chilloux J., Ottman N., Duparc T., Lichtenstein L. (2016). A purified membrane protein from Akkermansia muciniphila or the pasteurized bacterium improves metabolism in obese and diabetic mice. Nat. Med..

[24] Callahan B.J., Mcmurdie P.J., Rosen M.J., Han A.W., Johnson A.J.A., Holmes S.P. (2016). DADA_2_: High-resolution sample inference from Illumina amplicon data. Nat. Methods.

[25] Bolyen E., Rideout J.R., Dillon M.R., Bokulich N.A., Abnet C.C., Al-Ghalith G.A., Alexander H., Alm E.J., Arumugam M., Asnicar F. (2019). Reproducible, Interactive, Scalable and Extensible Microbiome Data Science using QIIME 2. Nat. Biotechnol..

[26] Liu R., Hong J., Xu X., Feng Q., Zhang D., Gu Y., Shi J., Zhao S., Liu W., Wang X. (2017). Gut microbiome and serum metabolome alterations in obesity and after weight-loss intervention. Nat. Med..

[27] Brestoff J.R., Artis D. (2013). Commensal bacteria at the interface of host metabolism and the immune system. Nat. Immunol..

[28] Husted A.S., Trauelsen M., Rudenko O., Hjorth S.A., Schwartz T.W. (2017). GPCR-Mediated Signaling of Metabolites. Cell Metab..

[29] Putignani L., Del Chierico F., Vernocchi P., Cicala M., Cucchiara S., Dallapiccola B. (2016). Gut Microbiota Dysbiosis as Risk and Premorbid Factors of IBD and IBS Along the Childhood–Adulthood Transition. Inflamm. Bowel Dis..

[30] Cummings J.H., Pomare E.W., Branch W.J., Naylor C.P., Macfarlane G.T. (1987). Short chain fatty acids in human large intestine, portal, hepatic and venous blood. Gut.

[31] Koh A., De Vadder F., Kovatcheva-Datchary P., Bäckhed F. (2016). From Dietary Fiber to Host Physiology: Short-Chain Fatty Acids as Key Bacterial Metabolites. Cell.

[32] Krishnan S., Ding Y., Saedi N., Choi M., Sridharan G.V., Sherr D.H., Yarmush M.L., Alaniz R.C., Jayaraman A., Lee K. (2018). Gut Microbiota-Derived Tryptophan Metabolites Modulate Inflammatory Response in Hepatocytes and Macrophages. Cell Rep..

[33] Bliss E.S., Whiteside E. (2018). The Gut-Brain Axis, the Human Gut Microbiota and Their Integration in the Development of Obesity. Front. Physiol..

[34] Neyrinck A.M., Van Hée V.F., Piront N., De Backer F., Toussaint O., Cani P.D., Delzenne N.M. (2012). Wheat-derived arabinoxylan oligosaccharides with prebiotic effect increase satietogenic gut peptides and reduce metabolic endotoxemia in diet-induced obese mice. Nutr. Diabetes.

[35] de Vos W.M., Tilg H., Van Hul M., Cani P.D. (2022). Gut microbiome and health: Mechanistic insights. Gut.

[36] Rastelli M., Cani P.D., Knauf C. (2019). The Gut Microbiome Influences Host Endocrine Functions. Endocr. Rev..

[37] Régnier M., Van Hul M., Knauf C., Cani P.D. (2021). Gut microbiome, endocrine control of gut barrier function and metabolic diseases. J. Endocrinol..

[38] Blaak E.E., Canfora E.E., Theis S., Frost G., Groen A.K., Mithieux G., Nauta A., Scott K., Stahl B., Van Harsselaar J. (2020). Short chain fatty acids in human gut and metabolic health. Benef. Microbes.

[39] Lin H., An Y., Tang H., Wang Y. (2019). Alterations of Bile Acids and Gut Microbiota in Obesity Induced by High Fat Diet in Rat Model. J. Agric. Food Chem..

[40] Tian Y., Gui W., Koo I., Smith P.B., Allman E.L., Nichols R.G., Rimal B., Cai J., Liu Q., Patterson A.D. (2020). The microbiome modulating activity of bile acids. Gut Microbes.

[41] Wang K., Liao M., Zhou N., Bao L., Ma K., Zheng Z., Wang Y., Liu C., Wang W., Wang J. (2019). Parabacteroides distasonis Alleviates Obesity and Metabolic Dysfunctions via Production of Succinate and Secondary Bile Acids. Cell Rep..

[42] Paganelli F.L., Luyer M., Hazelbag C.M., Uh H.W., Rogers M.R.C., Adriaans D., Berbers R.-M., Hendrickx A.P.A., Viveen M.C., Groot J.A. (2019). Roux-Y Gastric Bypass and Sleeve Gastrectomy directly change gut microbiota composition independent of surgery type. Sci. Rep..

[43] Sabate J.-M., Coupaye M., Ledoux S., Castel B., Msika S., Coffin B., Jouet P. (2016). Consequences of Small Intestinal Bacterial Overgrowth in Obese Patients Before and After Bariatric Surgery. Obes. Surg..

[44] Hibberd M.C., Wu M., Rodionov D.A., Li X., Cheng J., Griffin N.W., Barratt M.J., Giannone R.J., Hettich R.L., Osterman A.L. (2017). The effects of micronutrient deficiencies on bacterial species from the human gut microbiota. Sci. Transl. Med..

[45] Mach N., Clark A. (2017). Micronutrient Deficiencies and the Human Gut Microbiota. Trends Microbiol..

[46] Aron-Wisnewsky J., Prifti E., Belda E., Ichou F., Kayser B.D., Dao M.C., Verger E.O., Hedjazi L., Bouillot J.-L., Chevallier J.-M. (2018). Major microbiota dysbiosis in severe obesity: Fate after bariatric surgery. Gut.

[47] Tian P., Li B., He C., Song W., Hou A., Tian S., Meng X., Li K., Shan Y. (2016). Antidiabetic (type 2) effects of Lactobacillus G15 and Q14 in rats through regulation of intestinal permeability and microbiota. Food Funct..

[48] Tsai Y.-L., Lin T.-L., Chang C.-J., Wu T.-R., Lai W.-F., Lu C.-C., Lai H.-C. (2019). Probiotics, prebiotics and amelioration of diseases. J. Biomed. Sci..

[49] Foligné B., Parayre S., Cheddani R., Famelart M.-H., Madec M.-N., Plé C., Breton J., Dewulf J., Jan G., Deutsch S.-M. (2016). Immunomodulation properties of multi-species fermented milks. Food Microbiol..

[50] Hussain S.S., Bloom S.R. (2012). The regulation of food intake by the gut-brain axis: Implications for obesity. Int. J. Obes..

[51] Rastelli M., Van Hul M., Terrasi R., Lefort C., Régnier M., Beiroa D., Delzenne N.M., Everard A., Nogueiras R., Luquet S. (2020). Intestinal NAPE-PLD contributes to short-term regulation of food intake via gut-to-brain axis. Am. J. Physiol. Endocrinol. Metab..

[52] Schuster S.C. (2007). Next-generation sequencing transforms today’s biology. Nat. Methods.

