# Supplementary material for: Study of the Effect of Intestinal Microbes on Obesity: A Bibliometric Analysis
Source: Nutrients. 2023 Jul 23;15(14):3255. doi: 10.3390/nu15143255 (PMC10383578; doi:10.3390/nu15143255)

Table S1. Top 10 most local cited countries/regions related to the effect of intestinal microbes on obesity.

Country	Total citations	Citations per article
the United States	87,065	52.20
China	65,250	22.42
France	13,809	52.71
Canada	13,496	43.68
Spain	12,780	35.01
Belgium	11,648	107.85
Japan	10,641	32.25
Germany	10,155	59.04
Korea	9568	28.99
Netherlands	9542	53.61

Table S2. Top 25 burst keywords in articles related to the effect of intestinal microbes on obesity. Burst strength indicates the importance of keywords to the research field. The years between "Begin" and "End" represent the period when the keyword was more influential. The thick dark bar represents the year in which the burst keyword appeared and the burst duration.

Keywords	Year	Strength	Begin	End	2013 - 2022
ecology	2013	28.33	2013	2018	
human gut microbiota	2013	27.34	2013	2017	
microflora	2013	23.44	2013	2016	
diet induced obesity	2013	21.12	2013	2014	
inflammatory bowel disease	2013	15.88	2013	2018	
diversity	2013	13.38	2013	2015	
weight lo	2013	12.42	2013	2014	
escherichia coli	2013	11.85	2013	2017	
crohns disease	2013	11.39	2013	2016	
fecal microbiota	2013	11.39	2013	2017	
gene expression	2013	11.28	2013	2016	
endotoxemia	2013	10.82	2013	2016	
glucagon like peptide 1	2013	10.53	2013	2015	
bacteria	2013	10.14	2013	2015	
ulcerative coliti	2013	9.70	2013	2016	
flora	2013	9.55	2013	2014	
intestinal permeability	2014	12.25	2014	2016	
gastrointestinal tract	2014	10.65	2014	2017	
intestinal microbiota	2013	10.04	2014	2015	
irritable bowel syndrome	2015	16.00	2015	2018	
human gut microbiome	2016	12.08	2016	2018	
immune system	2017	12.76	2017	2019	

glucose homeostasis	2017	10.14	2017	2018	
obese patient	2019	9.30	2019	2022	
serum	2020	9.69	2020	2022	

Table S3. Top 25 burst references in articles related to the effect of intestinal microbes on obesity. Burst strength indicates the importance of references to the research field. The years between "Begin" and "End" represent the period when the reference was more influential. The thick dark bar represents the year in which the burst reference appeared and the burst duration.

References	Year	Strength	Begin	End	2013 - 2022
Qin JJ, 2012, NATURE, V490, P55, DOI 10.1038/nature11450, DOI	2012	90.81	2013	2017	
Turnbaugh PJ, 2009, NATURE, V457, P480, DOI 10.1038/nature07540, DOI	2009	86.15	2013	2014	
Wu GD, 2011, SCIENCE, V334, P105, DOI 10.1126/science.1208344, DOI	2011	77.55	2013	2016	
Qin JJ, 2010, NATURE, V464, P59, DOI 10.1038/nature08821, DOI	2010	72.8	2013	2015	
Caporaso JG, 2010, NAT METHODS, V7, P335, DOI 10.1038/nmeth.f.303, DOI	2010	71.81	2013	2015	
Schwiertz A, 2010, OBESITY, V18, P190, DOI 10.1038/oby.2009.167, DOI	2010	68.86	2013	2015	
Arumugam M, 2011, NATURE, V473, P174, DOI 10.1038/nature09944, DOI	2011	60.45	2013	2016	
Larsen N, 2010, PLOS ONE, V5, P0, DOI 10.1371/journal.pone.0009085, DOI	2010	53.15	2013	2015	
Yatsunenko T, 2012, NATURE, V486, P222, DOI 10.1038/nature11053, DOI	2012	51.79	2013	2017	
Vijay-Kumar M, 2010, SCIENCE, V328, P228, DOI 10.1126/science.1179721, DOI	2010	51.18	2013	2015	
Vrieze A, 2012, GASTROENTEROLOGY, V143, P913, DOI 10.1053/j.gastro.2012.06.031, DOI	2012	47.11	2013	2017	
Furet JP, 2010, DIABETES, V59, P3049, DOI 10.2337/db10-0253, DOI	2010	43.35	2013	2015	
De Filippo C, 2010, P NATL ACAD SCI USA, V107, P14691, DOI 10.1073/pnas.1005963107, DOI	2010	42.86	2013	2015	
Le Chatelier E, 2013, NATURE, V500, P541, DOI 10.1038/nature12506, DOI	2013	76.08	2014	2018	
Tremaroli V, 2012, NATURE, V489, P242, DOI 10.1038/nature11552, DOI	2012	65.85	2014	2017	
Karlsson FH, 2013, NATURE, V498, P99, DOI 10.1038/nature12198, DOI	2013	49.41	2014	2018	
Everard A, 2013, P NATL ACAD SCI USA, V110, P9066, DOI 10.1073/pnas.1219451110, DOI	2013	100.42	2015	2018	
David LA, 2014, NATURE, V505, P559, DOI 10.1038/nature12820, DOI	2014	75.51	2015	2019	

Ridaura VK, 2013, SCIENCE, V341, P1079, DOI 10.1126/science.1241214, DOI	2013	74.7	2015	2018	
Langille MGI, 2013, NAT BIOTECHNOL, V31, P814, DOI 10.1038/nbt.2676, DOI	2013	62.72	2016	2018	
Koh A, 2016, CELL, V165, P1332, DOI 10.1016/j.cell.2016.05.041, DOI	2016	47.58	2019	2022	
Callahan BJ, 2016, NAT METHODS, V13, P581, DOI 10.1038/NMETH.3869, DOI	2016	92.64	2020	2022	
Bolyen E, 2019, NAT BIOTECHNOL, V37, P852, DOI 10.1038/s41587-019-0209-9, DOI	2019	77.15	2020	2022	
Depommier C, 2019, NAT MED, V25, P1096, DOI 10.1038/s41591-019-0495-2, DOI	2019	63.61	2020	2022	
Wang K, 2019, CELL REP, V26, P222, DOI 10.1016/j.celrep.2018.12.028, DOI	2019	48.09	2020	2022	

Table S4. Top 10 most cited countries/regions related to the effect of intestinal microbes on obesity.

Country	Citations per article	Total Citations
Israel	124.18	6085
Belgium	107.85	11,648
Sweden	103.95	9148
Austria	76.65	2606
Germany	59.04	10,155
Netherlands	53.61	9542
France	52.71	13,809
Ireland	52.44	3304
the United States	52.20	87,065
Switzerland	51.27	2666

Figure S1. Country collaboration map. The red line indicates that there was collaboration between two countries. The more connections two countries have, the thicker the line is. The deeper the color of a country is, the more documents are published in this country.

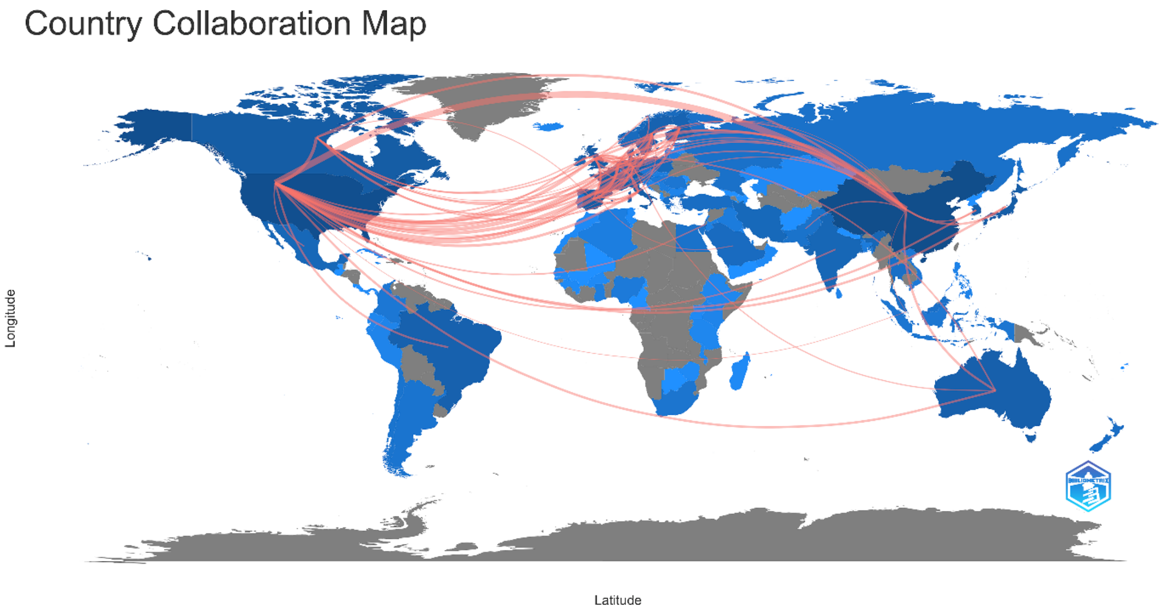

Figure S2. Dual-map overlay related to the effect of intestinal microbes on obesity.

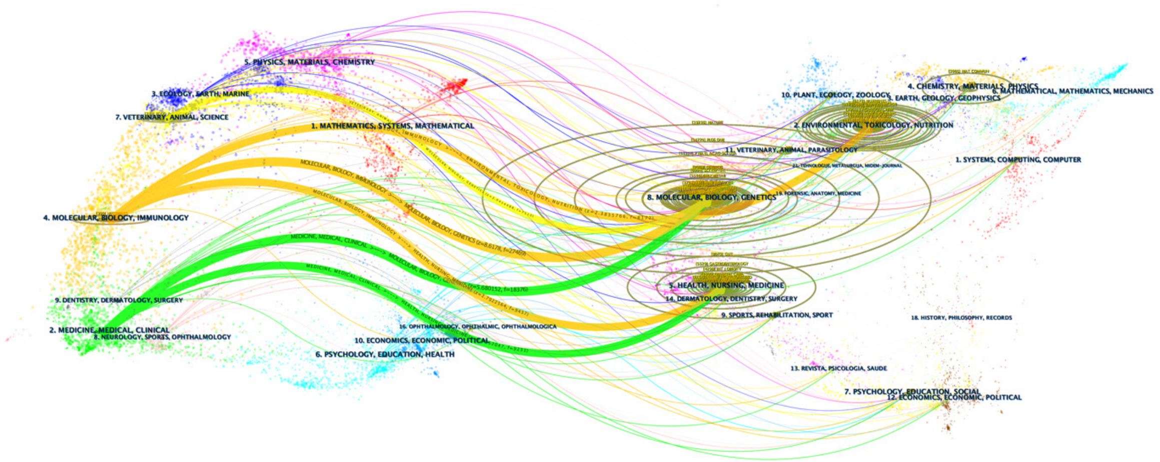

Figure S3. Cluster timeline view map of keyword analysis for the effect of intestinal microbes on obesity research.

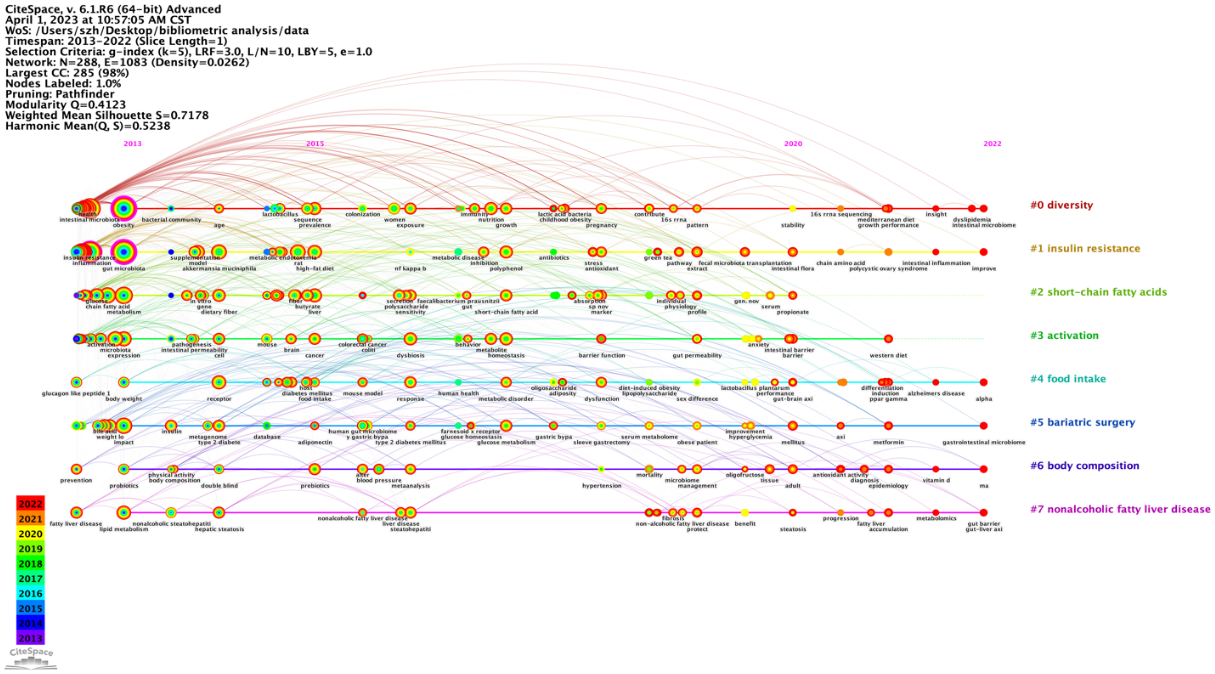

Supplement: Supplementary file 1 [file nutrients-15-03255-s001.zip › nutrients-2483950-supplementary.pdf]
